# Feeding practices and nutritional status of children age 6-23 months in Myanmar: A secondary analysis of the 2015-16 Demographic and Health Survey

**DOI:** 10.1371/journal.pone.0209044

**Published:** 2019-01-02

**Authors:** Kyaw Swa Mya, Aung Tin Kyaw, Thandar Tun

**Affiliations:** Department of Biostatistics and Medical Demography, University of Public Health, Yangon, Myanmar; University of Dhaka, BANGLADESH

## Abstract

Nutritional deficiencies are a major problem among developing countries including Myanmar. They can occur in all age groups, but the impact is more severe among children age 6–23 months as this period is critical for child development, and irreversible damages can occur due to nutritional deficiencies. Proper infant and young child feeding practices are pivotal to tackle nutritional problems and to prevent irreversible consequences among children. To assess the current feeding practices and associations with nutritional status, we conducted a secondary data analysis using the 2015–16 Myanmar Demographic and Health Survey. Multiple logistic regression analysis was done adjusting for covariates and the results were presented by adjusted odds ratios with 95% confidence intervals. A total of 1,222 children age 6–23 months were included in this analysis. Twenty percent were stunted and 43% were moderately anemic. Only 16% of children received a minimum acceptable diet, 25% received diverse food groups, 58% were fed with minimum meal frequency, 85% currently breastfed, and 59% consumed iron-rich foods. Breastfeeding reduced the odds of being stunted. Male sex, perceived small birth size, mother with short stature, and working mother were significant predictors of stunting. Iron-rich food consumption was inversely associated with moderate anemia. Male sex and maternal anemia were also significant predictors of moderate anemia. The study concluded that stunting and anemia among young children in Myanmar are major public health challenges that need urgent action. While further prospective research is needed to determine the effect of feeding practice on linear growth, interventions such as iron supplementation, and nutritional education programs according to the World Health Organization complementary feeding guidelines could help prevent stunting and childhood anemia and might reduce their prevalence in Myanmar.

## Introduction

Adequate nutrition is essential for growth and development of children, and malnutrition reflects poor social and economic development. Growth faltering results in adverse effects including poor physical and cognitive development, the impact of which may last a lifetime [1]. Short-term consequences include increased morbidity and mortality, developmental delay, and economic burden for sick children, while long-term consequences are stunted brains and stunted lives, hindering the development of entire societies. Hence, the period from conception to age 24 months is considered the “critical window” for the advancement of good growth, health, and behavioral and cognitive development [2].

Stunting, a sign of chronic under nutrition, is defined as the percentage of children whose height for age is below minus two standard deviations from the median of the World Health Organization (WHO) Child Growth Standards [3]. The prevalence of stunting among children under age 5 varies globally. In 2013, about half of the world’s 161 million stunted children lived in Asia, and over one-third in Africa [2]. About a quarter of the world’s children under age 5 live in South Asia and, among them, 38% have stunted growth [4]. In Myanmar, prevalence of stunting was 35% in 2009–2010 [5] and decreased to 29% in 2017 [6].

According to the WHO conceptual framework for childhood stunting, four main factors are responsible for stunting: (1) household and family factors—maternal disease, age, short stature, poor nutritional status, short birth intervals, poor care practices, inadequate water supply and sanitation, food insecurity, low caregiver education; (2) inadequate complementary feeding—poor-quality food, low dietary diversity and intake of food, infrequent and inadequate feeding, insufficient frequency of feeding; (3) inadequate practice of breastfeeding—early cessation of breastfeeding, non-exclusive breastfeeding; and (4) clinical and subclinical infection—diarrhea, malaria [7].

Childhood anemia is also a public health problem, with significant negative health consequences and adverse impacts on social and economic development [8]. A child is considered to be anemic if blood hemoglobin level is less than 11 g/dl, where 10–10.9 g/dl is mild anemia, 7–9.9 g/dl is moderate anemia, and less than 7 g/dl is severe anemia [9]. WHO estimates that nearly two-thirds of preschool children in Africa and Southeast Asia are anemic [10]. Severe anemia can cause child mortality. According to recent data from the Demographic and Health Surveys (DHS), the prevalence of anemia in preschool children is 53% in Nepal and 56% in Cambodia [11, 12]. In Myanmar its prevalence was 40% in 2011 [8] and 58% in 2015–16 [6], showing that prevalence of childhood anemia has increased. The prevalence of anemia is also above 50% in some other countries—including 56% in Bangladesh, 59% in India, and 61% in Pakistan—but is below 50% in some other developing countries—at 44% in Afghanistan, 36% in Sri Lanka, 35% in Philippines, and 32% in Indonesia [8].

Nutritional status of children under age 2 is highly influenced by feeding practices. To assess feeding practice precisely and to compare within and across nations, WHO recommends to use eight infant and young child feeding (IYCF) core indicators—early initiation of breastfeeding; exclusive breastfeeding for six months; continued breastfeeding at one year; introduction of solid, semi-solid, or soft foods; minimum dietary diversity; minimum meal frequency; minimum acceptable diet; and consumption of iron-rich or iron-fortified foods [13].

Although Myanmar established National Strategy on IYCF practices since 2011 [6], there are very limited studies on IYCF practices in Myanmar. Furthermore, few small studies have assessed the relationship between nutritional status of anemia and stunting with IYCF practices. Therefore, this study was conducted to explore the relationship between IYCF practices and nutritional status of children age 6–23 months, using the 2015–16 Myanmar DHS data.

## Materials and methods

### Data

Our study used data from the 2015–16 Myanmar DHS, which was nationally representative samples with 14 states and regions and the Nay Pyi Taw Union using two-stage sampling clusters (enumeration areas) as the primary sampling unit, and households as a secondary stage from which it sampled 13,260 households. Detail survey methods are described elsewhere [6]. In brief, the survey interviewed a total of 16,800 women and 7,500 men age 15–49 in the selected households. The data collection included taking blood samples and anthropometric measures of all children age 6–59 months and women age 15–49. The Myanmar DHS protocol was reviewed and approved by the Ethics Review Committee on Medical Research including Human Subjects in the Department of Medical Research, Ministry of Health and Sports, the Republic of the Union of Myanmar. Similarly, the survey protocol was approved by the ICF Institutional Review Board.

**Fig 1:**
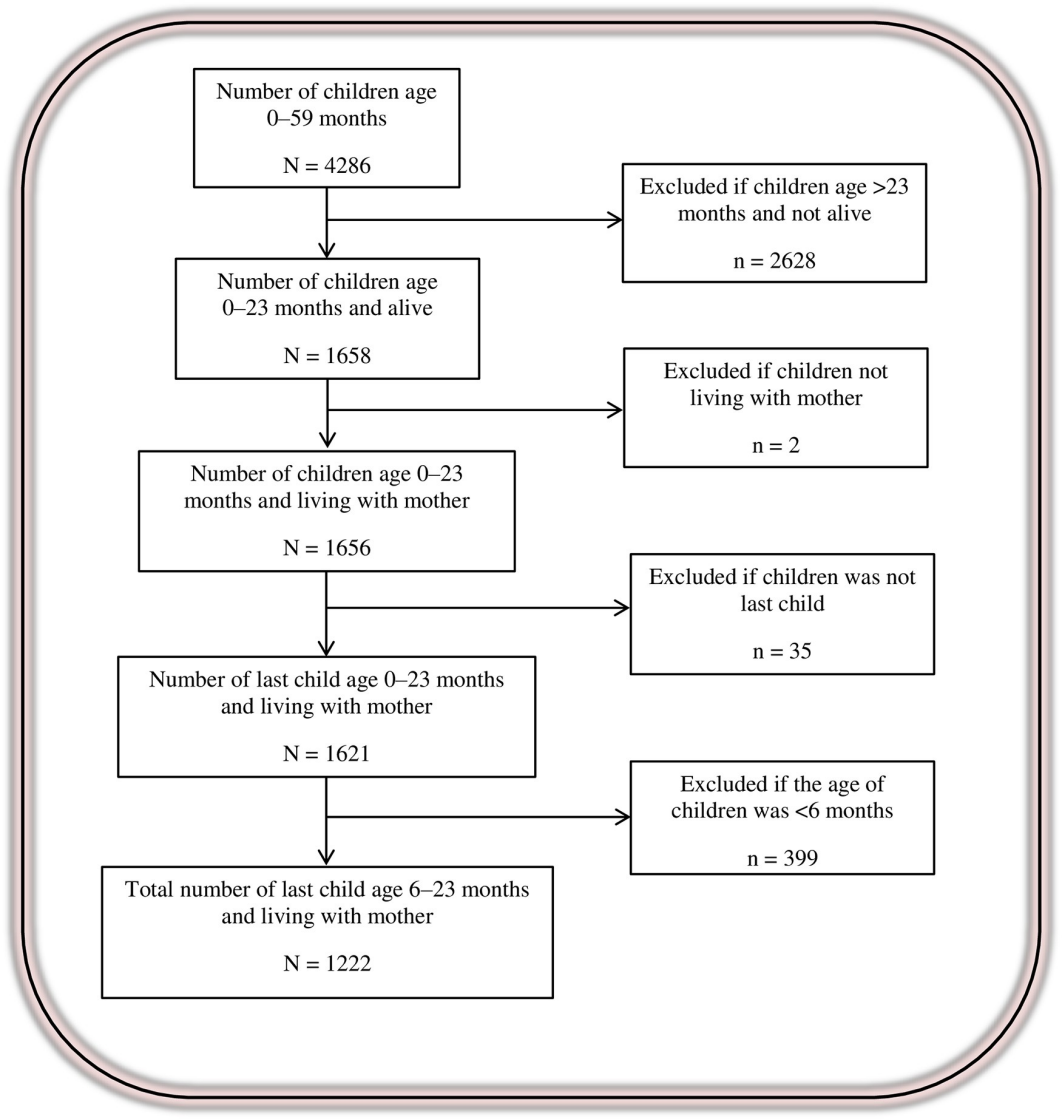
Population flow diagram (weighted number of cases).

We used Kids file (KR file) from Myanmar DHS data. From 4286 children age 0–59 months, we selected 1,222 last child age 6–23 months and living with their mother because information on complementary feeding practices was collected only for last child of this age group who lived together with interviewed mothers. Details of population flow were described in Fig 1.

### Variables

#### Dependent variables

We used stunting and anemia as dependent variables. Stunting was calculated from height-for-age Z-score using the WHO Child Growth Standards [14]. Children whose height-for-age Z-score is below minus two standard deviations (-2 SD) from the median of the reference population are considered short for their age (stunting), or chronically undernourished. We excluded missing values and biologically implausible values such as less than (-6 SD) and greater than (+6 SD) in our study [15]. The hemoglobin level of most of the children under age 24 months (about 76%) are less than 11 g/dl, however, they were apparently healthy. Hence we focused on moderate anemia, using cutoff value <10 g/dl to detect the association between IYCF practices and childhood anemia. We also excluded missing values and biologically implausible hemoglobin values such as less than 4 g/dl and greater than 18 g/dl from our analysis [9].

#### Independent variables

To assess children’s feeding practices, we used independent variables of five IYCF indicators—currently breastfed, minimum dietary diversity, minimum meal frequency, minimum acceptable diet, and consumption of iron-rich foods. These independent variables were recoded using information about foods given to the child in the last 24 hour before interview according to definitions of IYCF core indicators guidelines [13].

#### Covariates

This analysis considered characteristics of the child, the mother, and the household as covariates. Child’s characteristics included age, sex, and mother’s perceived birth size (size of child as reported subjectively by the respondent), birth order, immunization, deworming, vitamin A supplementation in the last six weeks before interview, and fever and diarrhea in the last two weeks before interview. Maternal characteristics included age, height, education, employment, number of antenatal care (ANC) visits, birth interval, and maternal anemia (<11 g/dl). Household characteristics included place of residence, region, family size, and household wealth status.

### Statistical analysis

We described background characteristics, the prevalence of stunting, moderate anemia and IYCF practices using frequency distribution tables. We used Pearson chi-square test for bivariate analysis. We assessed the association of IYCF practices with stunting and moderate anemia using multiple logistic regression analysis adjusting the covariates; child’s sex and perceived birth size, mother’s education, employment status, height, number of ANC visits and maternal anemia, and household’s residence and wealth status. We removed the region of residence variable from the analysis since we are not concerned with regional variation and also removed deworming. Multicollinearity was also tested among IYCF practices, showing that minimum dietary diversity and minimum acceptable diet were highly correlated (r = 0.75); hence, we removed minimum acceptable diet from the multiple logistic regression analysis. The results were presented using adjusted odds ratios (OR) with 95% CI.

While we adjusted each analysis that involved a hypothesis testing for the design effect using a predefined parameter established as a function of primary sampling unit (PSU), strata and sampling weights, we took into account sampling weight for frequency and percentage estimations for adjusting representativeness and nonresponse. All confidence intervals were calculated using standard error which was estimated by survey data analysis STATA command (SVY) adjusting for study design effect and nonresponses. We carried out all analyses on STATA (v.15.1), and p value <0.05 was set as a statistical significance.

## Results

The analysis included a total of 1,222 children age 6–23 months. Table 1 shows that 37% were age 12–17 months, 54% were male, 12% were below average in perceived birth size, and 36% were firstborn. Regarding immunization, only 31% completed full immunization at their age. Forty-nine percent of children received vitamin A supplementation and 22% received drugs for intestinal parasites in the last six months before interview. Previous history of fever was found in 22% of children, and diarrhea had occurred in 16% of children in the last two weeks before interview. Among the mothers, about half were age 20–29, 15% had no formal education, 59% were employed, 31% were short-statured (<150 cm), 10% did not receive ANC, 12% delivered their last child with a <24 months birth interval, and 46% were anemic. By characteristics of the household, 75% of the children resided in rural areas. About half of the children were from four states/regions—15% from Shan State, 14% from Ayeyarwaddy Region, 12% from Yangon Region, and 11% from Mandalay Region. About 32% of children were from large families of more than six members. Nearly half of children were from poor families (Table 1).

**Table 1:** Percent distribution of children age 6–23 months by child, mother and household characteristics (N = 1222).

Child’s characteristics	n (%)	Maternal characteristics	n (%)	Household characteristics	n (%)
Age in months		Age of mothers (Yrs)		Place of residence	
6–11	399 (32.6)	Less than 20	45 (3.7)	Urban	310 (25.4)
12–17	454 (37.2)	20–29	600 (49.1)	Rural	912 (74.6)
18–23	369 (30.2)	30–39	492 (40.2)	Region of residence	
Sex		40–47	85 (7.0)	Kachin	37 (3.1)
Male	658 (53.9)	Mother’s educational		Kayah	10 (0.8)
Female	564 (46.1)	No education	184 (15.1)	Kayin	47 (3.9)
Perceived birth size		Primary	547 (44.7)	Chin	16 (1.3)
Average/above	1033 (87.7)	Secondary	392 (32.1)	Sagaing	119 (9.7)
Below average	145 (12.3)	Higher	99 (8.1)	Taninthayi	34 (2.8)
Birth order		Mother’s employment status		Bago	105 (8.6)
1st child	434 (35.5)	Not working	504 (41.4)	Magway	83 (6.8)
2nd child	340 (27.9)	Working	714 (58.6)	Mandalay	133 (10.9)
3rd child	186 (15.2)	Mother’s height		Mon	38 (3.1)
4th and above	262 (21.4)	<150 cm	373 (31.0)	Rakhine	86 (7.1)
Immunization status		150–159 cm	739 (61.4)	Yangon	143 (11.7)
No/not complete	839 (68.7)	≥160 cm	91 (7.6)	Shan	180 (14.7)
Complete	383 (31.3)	Number of ANC visits		Ayeyarwaddy	168 (13.7)
Vitamin A in last six months		None	121 (10.0)	Naypyitaw	23 (1.8)
Not received	619 (50.7)	1–3	363 (30.1)	Family members	
Received	603 (49.3)	4 +	724 (59.9)	<5	361 (29.5)
Deworming in last six months		Birth interval group		5–6	475 (38.9)
No or don’t know	957 (78.3)	≥24 months	690 (88.3)	>6	386 (31.6)
Yes	265 (21.7)	<24 months	92 (11.7)	Wealth Index	
Fever in last two weeks		Maternal anemia (<11g/dl)		Poorest	327 (26.8)
No	956 (78.2)	No	642 (54.1)	Poorer	271 (22.2)
Yes	266 (21.8)	Yes	545 (45.9)	Middle	211 (17.2)
Diarrhea in last two weeks				Richer	215 (17.6)
No	1025 (83.9)			Richest	198 (16.2)
Yes	197 (16.1)				

### Prevalence of IYCF practices and nutritional status among children age 6–23 months

Table 2 shows the prevalence of IYCF practices. Most children age 6–23 months (85%) were still being breastfed, and 25% of children achieved minimum dietary diversity (at least four food groups). The percentage of children who achieved minimum required meal frequency was 58%, while only 16% achieved a minimum acceptable diet (apart from breast milk). Finally, 59% of children were fed iron-rich foods in the last 24 hours.

**Table 2:** The prevalence of IYCF practices and nutritional status among children age 6–23 months.

	Percent	95% CI
**IYCF practices**		
Currently breastfed	84.5	[81.7, 86.9]
Consumption of iron rich foods	58.5	[54.8, 62.2]
Minimum meal frequency	57.6	[54.3, 60.8]
Minimum dietary diversity	24.8	[21.7, 28.2]
Minimum acceptable diet	15.9	[13.5, 18.6]
**Nutritional status**		
^†^Stunting	20.2	[17.5, 23.3]
*Moderate anemia (<10g/dl)	42.8	[38.7, 47.2]

^†^98 cases were missing,

*282 cases were missing

### Prevalence of stunting by infant and young child feeding practices and child, maternal, and household characteristics

As Table 2 shows, prevalence of stunting among children age 6–23 months was 20%. Table 3 shows the prevalence of stunting by IYCF practices. Stunting was more prevalent in children who were not currently breastfed compared with those who were currently breastfed (30% versus 19%). However, stunting prevalence was higher in children who received minimum meal frequency compared with those who did not receive (23% versus 17%). The other IYCF practices were not significantly different in prevalence of stunting.

**Table 3:** Prevalence of stunting and moderate anemia by IYCF practices.

IYCF practices	Stunting		Moderate anemia	
	Percentage	95% CI	Percentage	95% CI
Currently breastfed		p = 0.004		p = 0.278
No	30.3	[22.7, 39.2]	37.6	[27.8, 48.5]
Yes	18.5	[15.6, 21.8]	43.8	[39.4, 48.2]
Minimum dietary diversity		p = 0.226		p = 0.563
No	19.2	[16.1, 22.8]	42.2	[37.6, 47.0]
Yes	23.3	[17.9, 29.7]	45.0	[36.6, 53.8]
Minimum meal frequency		p = 0.026		p = 0.701
No	16.5	[12.8, 20.9]	42.0	[35.8, 48.5]
Yes	23.0	[19.2, 27.2]	43.5	[38.4, 48.8]
Minimum acceptable diet		p = 0.896		p = 0.185
No	20.1	[17.1, 23.5]	41.7	[37.3, 46.2]
Yes	20.6	[14.8, 28.0]	49.1	[38.7, 59.6]
Consumption of iron rich foods		p = 0.64		p = 0.040
No	19.4	[15.4, 24.2]	48.0	[42.2, 53.9]
Yes	20.8	[17.3, 24.7]	39.6	[34.1, 45.4]

The results of bivariate analysis between stunting and other covariates were presented in S1 Table. Stunting was more prevalent in older (18–23 months) than younger (6–11 months) children, male than female children, and children below average perceived birth size compared with children of average and above perceived birth size. However, stunting was inversely associated with vitamin A supplementation. Stunting was also more common in children whose mothers were less educated, of short stature (<150 cm), and employed. Stunting was more prevalent in rural and poor families. There was significant regional variation i.e. highest in Kayah (33%) and lowest in Bago (9%). Details were described in S1 Table.

### Prevalence of moderate anemia by infant and young child feeding practices and child, mother and household characteristics

The prevalence of moderate anemia among children age 6–23 months was 43% (Table 2). Table 3 shows the prevalence of moderate anemia by IYCF practices. The prevalence of moderate anemia was higher in children who had not received iron-rich foods compared with those who had (48% versus 40%). Other IYCF practices were not associated with moderate anemia.

S1 Table presents results of bivariate analysis between moderate anemia and other covariates. Among child characteristics, only gender was significantly associated with moderate anemia—male children were more likely to be anemic than female children. By maternal characteristics, children of anemic mothers were more likely to be anemic than children of non-anemic mothers, while children whose mothers received no ANC visits had lower prevalence of anemia than those whose mothers received either two or three ANC visits, or the recommended four or more visits.

### Adjusted multiple logistic regressions: Stunting

Table 4 shows results of the adjusted multiple logistic regressions for stunting. The odds of stunting among currently breastfed children were 49% lower than for no breastfed children (aOR = 0.51; 95%CI 0.29, 0.88). Apart from this, no other IYCF practices were significantly associated with stunting. Among covariates, female children were less likely to be stunted than male children (aOR = 0.46; 95%CI 0.30, 0.71). Children with less than average perceived birth size had higher odds of stunting compared with children with average or above birth size (aOR = 2.38; 95%CI 1.44, 3.92). Children of working mothers were more likely to be stunted (aOR = 1.97; 95%CI 1.32, 2.94). Children of mothers whose height was greater than or equal to 150 cm had lower odds of stunting compared with children of mothers with height less than 150 cm (aOR = 0.42; 95%CI 0.27, 0.65 for mother’s height 150–159 cm) (aOR = 0.41; 95%CI 0.17, 0.94 for mother’s height ≥160 cm). Moreover, children from rural areas were more likely to be stunted compared with urban children (aOR = 2.08; 95%CI 1.15, 3.77).

**Table 4:** Association between IYCF practices and nutritional status (stunting and moderate anemia) among children age 6–23 months adjusting for covariates.

Variables	Stunting		Moderate anemia	
	AOR	95% CI	AOR	95% CI
**IYCF variables**				
Currently breastfed	0.51*	0.29–0.88	1.26	0.78–2.03
Minimum dietary diversity	0.92	0.57–1.47	1.37	0.88–2.13
Minimum meal frequency	1.40	0.90–2.18	1.18	0.82–1.68
Consumption of iron rich foods	1.20	0.74–1.96	0.66*	0.46–0.96
**Child’s characteristics**				
Sex of child (Female)	0.46***	0.30–0.71	0.62**	0.45–0.86
Perceived birth size (below average)	2.38***	1.44–3.92	1.10	0.66–1.83
**Maternal characteristics**				
Maternal age in year (Reference = 15–19)				
20–29	0.52	0.19–1.39	1.28	0.56–2.89
30–39	0.80	0.31–2.04	0.92	0.41–2.06
40–47	0.49	0.18–1.31	0.71	0.27–1.85
Maternal education (Reference = No education)				
Primary	0.75	0.43–1.29	0.81	0.49–1.32
Secondary	0.57	0.27–1.16	0.69	0.39–1.23
Higher	1.13	0.41–3.09	0.93	0.39–2.21
Mother’s employment status (Working)	1.97**	1.32–2.94	0.99	0.70–1.40
Mother’s height (Reference<150 cm)				
150–159 cm	0.42***	0.27–0.65	1.10	0.77–1.56
≥160 cm	0.41*	0.17–0.94	1.31	0.66–2.60
Number of ANC visits (Reference = None)				
1–3 times	1.42	0.76–2.65	2.20*	1.17–4.14
At least 4 times	1.28	0.64–2.53	2.37**	1.26–4.45
Maternal anemia (<11g/dl)	1.03	0.72–1.49	1.74***	1.27–2.39
**Household characteristics**				
Rural residence	2.08*	1.15–3.77	0.99	0.59–1.67
Wealth index (Reference = Poorest)				
Poorer	0.80	0.49–1.30	0.83	0.51–1.37
Middle	0.78	0.43–1.41	1.18	0.69–2.02
Richer	0.57	0.29–1.16	0.85	0.49–1.47
Richest	0.71	0.31–1.63	0.59	0.27–1.31

AOR = adjusted odds ratio,

***p<0.001,

**p<0.01,

*p<0.05

Covariates include child characteristics (sex, perceived birth weight), maternal characteristics (age, education, employment, height, number of ANC visits and maternal anaemia) and household characteristics (residence, wealth index).

### Adjusted multiple logistic regressions: Anemia

Table 4 also shows the results of the adjusted multiple logistic regressions for moderate anemia. The consumption of iron-rich foods was the only IYCF practice significantly associated with moderate anemia. The odds of moderate anemia among children who consumed iron-rich foods were 32% lower than those of children who did not consume them (aOR = 0.66; 95%CI 0.46, 0.96). Similar to stunting, moderate anemia was significantly associated with gender—female children were less likely to be anemic than male children (aOR = 0.62; 95%CI 0.45, 0.86). Maternal anemia was also associated with childhood anemia. The odds of moderate anemia among children of anemic mothers were 1.74 times higher than the odds among those of non-anemic mothers (aOR = 1.74; 95%CI 1.27, 2.39). However, the odds were higher among children of mothers who received either 1–3 ANC visits (aOR = 2.20; 95%CI 1.17, 4.14) or four or more ANC visits (aOR = 2.37; 95%CI 1.26, 4.45) compared with those of mothers who received no ANC visits.

## Discussion

This study examined the prevalence of stunting, moderate anemia, and IYCF practices among children age 6–23 months, and the association of IYCF practices with stunting and anemia. The findings show that among every five children, one was stunted and two were moderately anemic. Prevalence of all IYCF practices is low apart from breastfeeding, with less than one-fifth of children receiving a minimum acceptable diet and about half receiving adequate meal frequency and iron-rich food consumption. Only one-fourth of children have a diverse diet. The prevalence of stunting and anemia varies by background characteristics of children, mothers, and households. Stunting varies greatly among regions, from 9% to 32%. Children from rural and poor families are more likely to be stunted. As children’s age increases, the prevalence of stunting also increases and stunting is more common among male than female children. Stunting is also more common among children of mothers with short stature and mothers who are employed. Childhood anemia also varies by region, from 23% to 58%, but does not vary much by place of residence or wealth index. Similar to stunting, anemia is also more prevalent among male than female children. Maternal anemia is significantly associated with childhood anemia. Among IYCF practices, regression analysis showed that breastfeeding is significantly associated with lower odds of stunting, while consumption of iron-rich foods is inversely associated with childhood anemia.

The national estimate of stunting in Myanmar among children under age 5 is 29% [6]. Among those under age 2, a recent review of 137 developing countries reported a prevalence of stunting of 36% [16], which is above the prevalence of our study. A recent study in the Ayeyarwaddy region of Myanmar reported that stunting among children age 12–23 months was 35% [17], which is close to our regional estimate of stunting in Ayeyarwaddy (30%), and is consistent with regional variations in stunting prevalence. Our study found that stunting is more prevalent in rural areas, among poor families, male children, children of small birth size, mother with short stature, and working mothers. These findings are consistent with the findings of a study conducted in Ghana in 2011, which had found that child characteristics such as age, gender, reported size at childbirth, breastfeeding status, having diarrhea or fever in the preceding two weeks; household characteristics such as number of children in the household, child health insurance status, household wealth, ethnicity, religion and region were risk factors for under nutrition [18]. In our study, children still being breastfed were less likely to be stunted. Apart from this, other IYCF practices are not associated with stunting.

Our findings regarding IYCF practices are not consistent with other studies in the region. A study in Bangladesh on IYCF practice among children age 0–23 months found that children fed with adequate dietary diverse foods were negatively associated with stunting [19]. Another study in India reported that children who were not fed minimum meal frequency had 63% higher odds of being stunted, and that lower consumption of eggs was associated with increased odds of stunting in children age 6–23 months [20]. In contrast, another study in Sri Lanka stated that dietary diversity was not significantly associated with any anthropometric failure among children age 6–23 months [21]. Our study found a protective effect of breastfeeding on stunting, which might be due to the immune correlates found in the breast milk or to the fact that breast milk may reduce exposure to other environmental pathogens. In one systematic review, breastfeeding was associated with less occurrence of diarrhea and respiratory infections and as a result could avert hospital admissions [22].

For anemia, global prevalence for any anemia among children age 6–59 months was 42%. It varies greatly among the WHO regions- lowest in Western Pacific regions by 21.9% while highest in African region by 62.3% and Southeast Asian region accounted by 53.8% [8]. Hence, our estimates for children age 6–59 months (58%) and for children 6–23 months (76% for all anemia and 43% for moderate anemia) are higher than global and regional prevalence of children age 6–59 months. However, our estimates are lower than in the study in Myanmar by Hlaing and colleagues (2015), which found any anemia prevalence among children age 1–2 to be 88% [23]. A global review of anemia reported that anemia prevalence is highest among children under age 1 followed by age 1–4 compared with other age categories. Moreover this trend did not change favorably from 1990 to 2010 [24].

Our study shows that the prevalence of anemia among children who consumed iron-rich foods is significantly lower than that of children who did not consume them. Moreover, mothers who attended four or more ANC visits also fed their children more iron-rich foods (S2 Table). These findings are not consistent with a Bangladesh study of anemia among children age 6–11 months and feeding practices, which showed that there was no association between anemia and previous-day consumption of iron-rich food and consumption of a minimum acceptable diet [25]. Another study in Laos found no association between anemia and breastfeeding [26].

The possible reason for the finding of no association of IYCF practices with stunting and moderate anemia in our study is that the DHS survey asked about feeding practices in the last 24 hours before interview and assumed it to be the usual dietary pattern. However, it might not be representative for all children because food consumption might vary day by day [27]. Although IYCF indicators are simple and effective in assessing complementary feeding practices for large-scale surveys, the level of sensitivity and specificity of these indicators on dietary quality should be considered in assessing association of IYCF with the linear growth of children [28].

Although a study conducted in Myanmar stated that iron deficiency was the main cause of anemia, the biomarkers recommended by WHO for iron status in populations were not used in their study [23]. Our study could not provide the evidence of low iron levels among children with anemia, as the DHS does not collect biomarkers for iron status. Hence further research is needed to explore the main causes of anemia. Our study also found higher prevalence of childhood anemia among children of mothers who attended either one to three ANC visits or four and more compared with mothers with no ANC visits. This association might be because the DHS survey was cross-sectional, and in our analysis we were unable to control all possible confounders and their mediation. The association of ANC visits with childhood anemia may have been more indirect than direct, since anemia was not assessed directly after birth but rather when the child was older. Another possibility is that women who make more ANC visits are women who may also be more anemic themselves.

IYCF practices of Myanmar are poor among South/Southeast Asian countries. Regionally, Myanmar’s IYCF practices of minimum meal frequency, diverse food consumption, and minimum acceptable diet are lower than in Nepal, Cambodia, and Indonesia, and more or less similar to Pakistan, Afghanistan, and Bangladesh. Iron-rich food consumption in Myanmar is lower than in Cambodia and Indonesia but higher than in Bangladesh, Nepal, and India [11, 12, 29–33]. Many factors influence IYCF practices. Among them, maternal characteristics of education and ANC visits were found to be far more important than other household variables such as family size, wealth index, and child characteristics. The study found that the higher the level of maternal education, the better the IYCF practices. Similarly, IYCF practices among mothers who attended four or more ANC visits were better than among mothers who attended fewer than four ANC visits. Details are presented in S2 Table. Hence improving female education and offering universal ANC services accompanied with community-based nutritional education could be an alternative way to improve IYCF practices.

This study was the first study to assess the association between IYCF practices and nutritional status of children age 6–23 months at the national level in Myanmar. The study findings are nationally representative. All the analyses accounted for the cluster survey design, and the regression results were presented after adjusting the covariates. As Myanmar DHS 2015–16 was a cross-sectional study, our findings cannot make any claims about the causality of the associations. Although IYCF practices were assessed by asking mothers about the types of liquids and foods the child consumed during the previous day or night (last 24 hours) before interview, respondent’s recall and reporting bias could have possibly influenced the results. We assumed that the practices reported by mothers are more or less the same throughout the children’s life and assessed their association with stunting and anemia. Hence, if the reported practices and actual practices were different, it would influence the study’s findings. All of the IYCF practices were calculated by combining information from more than one variable; as a result, the practices will be under- or over-estimated depending on the accuracy of the reported practices.

## Conclusions

The study concluded that stunting and anemia among children age 6–23 months in Myanmar are major public health challenges that require urgent action. Children should be fed with diverse food groups including iron-rich foods according to WHO complementary feeding guidelines. Continued breastfeeding to age 2 and beyond should also be promoted. Since stunting begins in the prenatal period, nutritional promotion for pregnant mothers and early and regular ANC visits should be encouraged. While further prospective research is needed to determine the effect of feeding practices on linear growth, interventions such as iron supplementation and nutritional education programs could help prevent stunting and childhood anemia and might reduce their prevalence in Myanmar.

## Supporting information

S1 Table Prevalence of stunting and moderate anemia among children 6–23 months by child, maternal and household characteristics. (DOCX)

S2 Table Prevalence of IYCF practices by child, maternal and household characteristics. (DOCX)
