# Supplementary material for: Feeding practices and nutritional status of children age 6-23 months in Myanmar: A secondary analysis of the 2015-16 Demographic and Health Survey
Source: PLoS One. 2019 Jan 2;14(1):e0209044. doi: 10.1371/journal.pone.0209044 (PMC6314612; doi:10.1371/journal.pone.0209044)
Supplement: S1 Table — (DOCX) [file pone.0209044.s001.docx]

**S1 Table. Prevalence of stunting and moderate anemia among children 6-23 months by child, maternal and household characteristics**

|  | **Stunt** | | |  | **Moderate anemia** | | |
| --- | --- | --- | --- | --- | --- | --- | --- |
|  | **%** | **CI** | **p value** |  | **%** | **CI** | **p value** |
| **Child's characteristics** |  |  |  |  |  |  |  |
| **Age** |  |  | <0.001 |  |  |  | 0.318 |
| 6-11 months | 14.2 | [10.5,18.8] |  |  | 42.8 | [36.4,49.4] |  |
| 12-17 months | 15.8 | [12.0,20.6] |  |  | 46.0 | [39.9,52.1] |  |
| 18-23 months | 32.1 | [26.3,38.4] |  |  | 39.3 | [32.3,46.7] |  |
| **Sex** |  |  | <0.001 |  |  |  | 0.001 |
| Male | 26.0 | [21.8,30.6] |  |  | 48.9 | [43.6,54.2] |  |
| Female | 13.7 | [10.5,17.7] |  |  | 36.1 | [30.3,42.4] |  |
| **Perceived birth size** |  |  | <0.001 |  |  |  | 0.850 |
| Average and above | 18.1 | [15.2,21.5] |  |  | 42.8 | [38.3,47.3] |  |
| Below average | 33.4 | [25.3,42.6] |  |  | 43.9 | [33.0,55.4] |  |
| **Birth order** |  |  | 0.139 |  |  |  | 0.774 |
| 1st child | 17.8 | [13.7,22.7] |  |  | 44.9 | [38.2,51.7] |  |
| 2nd child | 19.0 | [14.4,24.7] |  |  | 43.2 | [36.1,50.6] |  |
| 3rd child | 18.8 | [12.2,27.8] |  |  | 40.3 | [31.9,49.4] |  |
| 4th and above | 26.6 | [20.6,33.7] |  |  | 40.7 | [33.9,47.9] |  |
| **Immunization status** |  |  | 0.109 |  |  |  | 0.917 |
| No/not complete immunization | 21.7 | [18.5,25.3] |  |  | 42.7 | [38.2,47.4] |  |
| Complete immunization | 16.9 | [12.7,22.2] |  |  | 43.2 | [35.9,50.7] |  |
| **Vitamin A in last 6 months** |  |  | 0.008 |  |  |  | 0.241 |
| Not received/not known | 16.3 | [12.9,20.4] |  |  | 45.1 | [39.8,50.6] |  |
| Received | 24.2 | [20.1,28.8] |  |  | 40.7 | [35.1,46.6] |  |
| **Deworming in last 6 months** |  |  | 0.099 |  |  |  | 0.692 |
| No or don't know | 18.8 | [15.8,22.2] |  |  | 43.3 | [38.7,48.0] |  |
| Yes | 25.4 | [18.7,33.5] |  |  | 41.5 | [33.4,50.0] |  |
| **Fever in last two weeks** |  |  | 0.635 |  |  |  | 0.572 |
| No | 20.6 | [17.5,24.0] |  |  | 43.4 | [38.8,48.2] |  |
| Yes | 18.8 | [13.3,26.0] |  |  | 41.0 | [33.6,48.9] |  |
| **Diarrhea in last two weeks** |  |  | 0.351 |  |  |  | 0.336 |
| No | 20.8 | [17.7,24.2] |  |  | 42.0 | [37.3,46.9] |  |
| Yes | 17.2 | [11.6,24.7] |  |  | 47.3 | [38.0,56.7] |  |
| **Maternal characteristics** |  |  |  |  |  |  | |
| **Age of mothers (Yrs)** |  |  | 0.138 |  |  |  | 0.284 |
| Less than 20 | 31.1 | [16.9,50.0] |  |  | 36.8 | [21.4,55.4] |  |
| 20-29 | 17.3 | [13.9,21.2] |  |  | 46.3 | [40.4,52.4] |  |
| 30-39 | 22.6 | [18.1,27.8] |  |  | 39.7 | [33.8,45.9] |  |
| 40-47 | 21.6 | [13.2,33.3] |  |  | 38.6 | [26.5,52.2] |  |
| **Mother's educational level** |  |  | <0.001 |  |  |  | 0.667 |
| No education | 31.6 | [24.4,39.8] |  |  | 41.5 | [31.2,52.6] |  |
| Primary | 22.2 | [18.0,27.1] |  |  | 44.6 | [39.0,50.3] |  |
| Secondary | 12.4 | [8.9,17.0] |  |  | 39.9 | [33.4,46.8] |  |
| Higher | 18.8 | [10.9,30.2] |  |  | 46.5 | [33.2,60.4] |  |
| **Mother's employment status** |  |  | <0.001 |  |  |  | 0.739 |
| Not working | 14.2 | [10.8,18.3] |  |  | 43.6 | [37.6,49.7] |  |
| Working | 24.7 | [20.8,29.1] |  |  | 42.3 | [37.1,47.7] |  |
| **Mother's height** |  |  | <0.001 |  |  |  | 0.910 |
| < 150 cm | 30.7 | [24.8,37.3] |  |  | 43.9 | [37.3,50.7] |  |
| 150 - 159 cm | 15.8 | [12.7,19.5] |  |  | 42.3 | [37.0,47.7] |  |
| ≥ 160 cm | 13.3 | [7.0,23.9] |  |  | 44.1 | [30.5,58.7] |  |
| **Number of AN visits** |  |  | 0.079 |  |  |  | 0.025 |
| None | 20.9 | [13.6,30.5] |  |  | 28.2 | [20.5,37.3] |  |
| 1-3 | 24.4 | [19.3,30.5] |  |  | 45.2 | [38.3,52.4] |  |
| 4 | 17.5 | [14.2,21.4] |  |  | 43.1 | [38.1,48.4] |  |
| **Birth interval group** |  |  | 0.136 |  |  |  | 0.509 |
| ≥24 months | 20.4 | [16.7,24.6] |  |  | 42.0 | [37.1,47.1] |  |
| <24 months | 29.2 | [18.9,42.2] |  |  | 37.2 | [25.5,50.7] |  |
| **Maternal anemia (<11g/dl)** |  |  | 0.647 |  |  |  | <0.001 |
| No | 19.9 | [16.5,23.8] |  |  | 36.0 | [30.9,41.4] |  |
| Yes | 21.1 | [17.2,25.6] |  |  | 50.7 | [44.9,56.5] |  |
| **Household characteristics** |  |  |  |  |  | | |
| **Place of residence** |  |  | <0.001 |  |  |  | 0.421 |
| Urban | 10.4 | [7.0,15.3] |  |  | 39.8 | [31.4,48.7] |  |
| Rural | 23.4 | [20.1,27.0] |  |  | 43.9 | [39.1,48.8] |  |
| **Region of residence** |  |  | 0.002 |  |  |  | 0.053 |
| Kachin | 15.0 | [8.9,24.2] |  |  | 45.7 | [33.8,58.0] |  |
| Kayah | 32.7 | [23.8,43.1] |  |  | 36.6 | [27.9,46.3] |  |
| Kayin | 14.1 | [8.2,23.2] |  |  | 37.2 | [28.5,46.9] |  |
| Chin | 27.9 | [20.5,36.8] |  |  | 39.7 | [30.9,49.2] |  |
| Sagaing | 21.4 | [13.3,32.6] |  |  | 47.7 | [34.3,61.4] |  |
| Taninthayi | 11.4 | [4.9,24.1] |  |  | 38.6 | [30.2,47.8] |  |
| Bago | 9.2 | [4.3,18.9] |  |  | 39.3 | [26.4,53.8] |  |
| Magway | 14.8 | [6.2,31.5] |  |  | 58.1 | [44.6,70.4] |  |
| Mandalay | 25.1 | [15.4,38.2] |  |  | 33.1 | [21.6,47.0] |  |
| Mon | 18.5 | [10.3,30.9] |  |  | 49.0 | [36.1,62.1] |  |
| Rakhine | 20.8 | [14.5,28.8] |  |  | 52.4 | [38.0,66.4] |  |
| Yangon | 9.5 | [4.6,18.8] |  |  | 41.4 | [28.2,55.9] |  |
| Shan | 29.7 | [20.4,41.1] |  |  | 23.4 | [13.5,37.5] |  |
| Ayeyarwaddy | 30.7 | [22.4,40.5] |  |  | 49.2 | [35.6,62.9] |  |
| Naypyitaw | 13.9 | [8.0,23.0] |  |  | 50.7 | [30.1,71.1] |  |
| **Family members** |  |  | 0.084 |  |  |  | 0.198 |
| <5 | 15.7 | [11.9,20.6] |  |  | 45.0 | [37.5,52.8] |  |
| 5-6 | 22.3 | [18.1,27.2] |  |  | 38.5 | [33.1,44.2] |  |
| >6 | 21.9 | [17.2,27.3] |  |  | 46.2 | [38.9,53.6] |  |
| **Wealth Index** |  |  | 0.023 |  |  |  | 0.378 |
| Poorest | 26.3 | [20.8,32.7] |  |  | 43.8 | [35.8,52.1] |  |
| Poorer | 20.7 | [15.4,27.1] |  |  | 42.6 | [35.1,50.5] |  |
| Middle | 21.5 | [16.1,28.1] |  |  | 50.0 | [40.5,59.5] |  |
| Richer | 13.2 | [8.3,20.4] |  |  | 40.7 | [32.2,49.9] |  |
| Richest | 14.9 | [9.3,23.1] |  |  | 35.7 | [25.6,47.2] |  |
| **Total** | **20.2** | **[17.5,23.3]** |  |  | **42.8** | **[38.7,47.2]** |  |
