# Supplementary material for: Feeding practices and nutritional status of children age 6-23 months in Myanmar: A secondary analysis of the 2015-16 Demographic and Health Survey
Source: PLoS One. 2019 Jan 2;14(1):e0209044. doi: 10.1371/journal.pone.0209044 (PMC6314612; doi:10.1371/journal.pone.0209044)
Supplement: S2 Table — (DOCX) [file pone.0209044.s002.docx]

**S2 Table. Prevalence of IYCF practices by child, maternal and household characteristics**

|  | **Breastfed** | | **Minimum dietary diversity** | | **Minimum meal frequency** | | **Minimum acceptable diet** | | **Iron rich foods** | |
| --- | --- | --- | --- | --- | --- | --- | --- | --- | --- | --- |
|  | **%** | **95% CI** | **%** | **95% CI** | **%** | **95% CI** | **%** | **95% CI** | **%** | **95% CI** |
| **Child's characteristics** | | | | | | | | | | |
| **Age** |  | <0.001 |  | <0.001 |  | 0.051 |  | 0.004 |  | <0.001 |
| 6-11 months | 97.0 | [94.6,98.4] | 12.6 | [9.0,17.2] | 55.1 | [50.0,60.0] | 9.9 | [6.7,14.4] | 37.4 | [31.9,43.2] |
| 12-17 months | 87.1 | [82.5,90.6] | 27.8 | [22.9,33.3] | 55.1 | [49.9,60.3] | 19.2 | [15.1,24.1] | 65.5 | [59.7,70.9] |
| 18-23 months | 67.9 | [61.4,73.7] | 34.2 | [28.8,40.0] | 63.3 | [57.2,69.0] | 18.1 | [14.1,22.9] | 72.6 | [67.2,77.4] |
| **Sex** |  | 0.087 |  | 0.022 |  | 0.55 |  | 0.736 |  | 0.704 |
| Male | 82.7 | [79.0,85.9] | 27.8 | [23.4,32.8] | 56.7 | [51.9,61.3] | 16.2 | [13.1,19.9] | 59.1 | [54.3,63.7] |
| Female | 86.6 | [82.9,89.6] | 21.2 | [17.6,25.3] | 58.7 | [54.1,63.1] | 15.4 | [12.2,19.3] | 57.8 | [52.7,62.9] |
| **Perceived birth size** |  | 0.937 |  | 0.423 |  | 0.758 |  | 0.153 |  | 0.041 |
| Average /above | 84.1 | [81.1,86.8] | 25.5 | [22.2,29.0] | 57.9 | [54.1,61.5] | 16.6 | [14.0,19.6] | 59.7 | [55.7,63.5] |
| Below average | 83.9 | [75.7,89.6] | 22.0 | [15.1,31.1] | 56.3 | [46.8,65.3] | 11 | [6.1,18.9] | 49.6 | [40.3,58.8] |
| **Birth order** |  | 0.215 |  | 0.031 |  | 0.572 |  | 0.06 |  | 0.008 |
| 1st child | 81.7 | [76.8,85.8] | 28.8 | [23.8,34.4] | 57.0 | [51.8,61.9] | 19.7 | [15.4,24.7] | 64.5 | [59.0,69.6] |
| 2nd child | 87.5 | [82.5,91.2] | 25.2 | [20.3,30.7] | 58.7 | [52.3,64.8] | 15.7 | [11.7,20.7] | 58.8 | [52.2,65.1] |
| 3rd child | 82.6 | [74.4,88.5] | 24.8 | [18.4,32.6] | 61.6 | [53.4,69.2] | 13.7 | [8.9,20.5] | 56.8 | [48.5,64.8] |
| 4th and above | 86.6 | [81.6,90.5] | 17.6 | [12.8,23.7] | 54.3 | [47.1,61.4] | 11.2 | [7.7,16.1] | 49.3 | [42.0,56.7] |
| **Immunization status** |  | 0.373 |  | 0.285 |  | 0.249 |  | 0.432 |  | 0.105 |
| No/not complete | 83.7 | [80.2,86.6] | 23.7 | [20.1,27.8] | 56.2 | [52.4,60.0] | 15.2 | [12.5,18.3] | 56.7 | [52.5,60.9] |
| Complete | 86.4 | [81.1,90.4] | 27.1 | [22.1,32.8] | 60.6 | [54.2,66.7] | 17.4 | [13.0,22.8] | 62.4 | [56.2,68.2] |
| **Vitamin A in last 6 months** | | 0.007 |  | 0.012 |  | 0.021 |  | 0.033 |  | <0.001 |
| Not received/not known | 88.0 | [84.3,90.9] | 20.8 | [16.7,25.6] | 53.7 | [48.9,58.5] | 13.2 | [10.2,16.8] | 51.7 | [46.3,57.1] |
| Received | 81.0 | [76.7,84.6] | 28.9 | [24.6,33.5] | 61.6 | [57.0,65.9] | 18.6 | [15.1,22.8] | 65.5 | [60.9,69.8] |
| **Deworming in last 6 months** | | 0.005 |  | <0.001 |  | 0.133 |  | 0.002 |  | <0.001 |
| No or don't know | 86.5 | [83.7,88.8] | 22.1 | [18.9,25.8] | 56.2 | [52.4,59.9] | 14.0 | [11.5,16.8] | 55.3 | [51.1,59.4] |
| Yes | 77.5 | [69.8,83.6] | 34.3 | [28.3,40.9] | 62.8 | [55.1,69.9] | 22.6 | [17.3,29.0] | 70.1 | [63.0,76.3] |
| **Fever in last two weeks** |  | 0.004 |  | 0.588 |  | 0.975 |  | 0.72 |  | 0.56 |
| No | 82.8 | [79.4,85.7] | 25.2 | [21.6,29.2] | 57.6 | [53.9,61.2] | 15.6 | [13.0,18.6] | 58.0 | [53.8,62.1] |
| Yes | 90.7 | [86.3,93.8] | 23.3 | [18.1,29.4] | 57.7 | [51.4,63.8] | 16.6 | [12.2,22.2] | 60.3 | [53.2,67.1] |
| **Diarrhea in last two weeks** | | 0.431 |  | 0.184 |  | 0.744 |  | 0.405 |  | 0.852 |
| No | 84.9 | [81.9,87.4] | 23.9 | [20.6,27.6] | 57.4 | [53.7,60.9] | 15.4 | [12.8,18.4] | 58.4 | [54.1,62.5] |
| Yes | 82.7 | [76.4,87.5] | 29.1 | [22.3,37.0] | 58.8 | [50.7,66.5] | 18.1 | [12.8,25.1] | 59.2 | [51.3,66.7] |
| **Maternal characteristics** | | | | | | | | | | |
| **Age of mothers (Yrs)** |  | 0.104 |  | 0.313 |  | 0.302 |  | 0.549 |  | 0.005 |
| Less than 20 | 74.7 | [56.2,87.2] | 12.1 | [4.5,28.7] | 56.1 | [39.5,71.5] | 10.3 | [3.4,27.3] | 37.0 | [22.4,54.5] |
| 20-29 | 82.9 | [78.9,86.3] | 25.4 | [21.0,30.3] | 54.6 | [50.5,58.7] | 15.3 | [12.1,19.3] | 62.2 | [57.0,67.1] |
| 30-39 | 87.7 | [83.7,90.9] | 25.8 | [21.6,30.4] | 60.7 | [55.2,65.9] | 17.5 | [14.2,21.4] | 58.3 | [52.8,63.5] |
| 40-47 | 82.4 | [71.7,89.6] | 21.6 | [13.4,32.9] | 61.5 | [49.2,72.5] | 12.9 | [6.8,23.1] | 45.4 | [33.5,57.8] |
| **Mother's educational level** | | 0.723 |  | <0.001 |  | 0.665 |  | 0.009 |  | <0.001 |
| No education | 83.6 | [75.0,89.7] | 15.0 | [8.7,24.6] | 53.2 | [45.8,60.5] | 9.8 | [5.1,18.1] | 45.2 | [35.3,55.5] |
| Primary | 85.9 | [81.7,89.2] | 21.4 | [17.6,25.7] | 58.1 | [52.9,63.1] | 13.6 | [10.7,17.2] | 54.1 | [48.8,59.3] |
| Secondary | 83.9 | [79.1,87.8] | 27.8 | [22.9,33.3] | 58.0 | [52.3,63.4] | 19.2 | [15.2,24.0] | 66.8 | [61.4,71.7] |
| Higher | 80.9 | [70.2,88.3] | 49.8 | [38.2,61.4] | 61.5 | [48.6,72.9] | 26.1 | [17.5,36.9] | 75.1 | [62.9,84.3] |
| **Mother's employment status** | | 0.077 |  | 0.026 |  | 0.001 |  | 0.096 |  | 0.57 |
| Not working | 87.0 | [82.7,90.3] | 20.7 | [16.6,25.5] | 50.6 | [45.2,56.0] | 13.3 | [10.0,17.4] | 57.4 | [52.0,62.6] |
| Working | 82.7 | [79.3,85.6] | 27.5 | [23.4,32.0] | 62.5 | [58.3,66.5] | 17.5 | [14.4,21.1] | 59.3 | [54.5,64.0] |
| **Mother's height** |  | 0.982 |  | 0.104 |  | 0.687 |  | 0.021 |  | 0.089 |
| <150 cm | 84.7 | [80.1,88.4] | 24.6 | [19.5,30.5] | 58.5 | [53.1,63.8] | 15.0 | [11.5,19.5] | 54.2 | [47.7,60.5] |
| 150 - 159 cm | 84.3 | [80.9,87.2] | 23.1 | [19.7,26.8] | 56.3 | [51.8,60.7] | 14.5 | [11.7,17.9] | 59.4 | [55.0,63.7] |
| ≥160 cm | 84.8 | [73.4,91.9] | 34.5 | [24.2,46.5] | 61.2 | [48.1,72.9] | 27.0 | [17.8,38.9] | 68.5 | [55.9,78.9] |
| **Number of AN visits** |  | 0.729 |  | 0.003 |  | 0.085 |  | <0.001 |  | <0.001 |
| None | 86.6 | [79.0,91.8] | 13.0 | [7.3,22.2] | 46.5 | [36.6,56.7] | 3.5 | [1.2,9.9] | 41.9 | [30.8,53.8] |
| 1-3 | 85.8 | [80.6,89.8] | 20.4 | [15.2,26.8] | 58.3 | [52.2,64.0] | 12.6 | [8.8,17.8] | 53.3 | [47.4,59.1] |
| 4 | 84.1 | [80.5,87.2] | 28.5 | [24.9,32.3] | 58.9 | [54.7,63.1] | 19.7 | [16.7,23.1] | 63.4 | [59.1,67.5] |
| **Birth interval group** |  | 0.169 |  | 0.084 |  | 0.485 |  | 0.093 |  | 0.083 |
| ≥24 months | 87.0 | [83.7,89.7] | 23.3 | [19.8,27.2] | 58.1 | [53.7,62.3] | 14.7 | [11.9,18.0] | 56.2 | [51.6,60.8] |
| <24 months | 81.2 | [69.3,89.2] | 14.8 | [8.5,24.5] | 53.8 | [42.3,64.9] | 7.1 | [2.9,16.3] | 45.1 | [33.3,57.6] |
| **Maternal anemia (<11g/dl)** | | 0.069 |  | 0.18 |  | 0.061 |  | 0.552 |  | 0.144 |
| No | 82.3 | [78.4,85.6] | 26.0 | [21.8,30.7] | 60.4 | [56.0,64.6] | 16 | [12.8,19.9] | 60.9 | [56.0,65.5] |
| Yes | 86.9 | [82.9,90.0] | 22.3 | [18.5,26.5] | 53.7 | [48.4,59.0] | 14.7 | [11.7,18.3] | 55.6 | [50.0,61.1] |
| **Household characteristics** | | | | | | | | | | |
| **Place of residence** |  | 0.043 |  | 0.001 |  | 0.113 |  | 0.023 |  | 0.001 |
| Urban | 80.1 | [74.3,84.9] | 33.9 | [27.5,40.9] | 53.0 | [46.3,59.6] | 20.8 | [15.9,26.7] | 69.0 | [61.7,75.5] |
| Rural | 86 | [82.8,88.7] | 21.7 | [18.3,25.5] | 59.1 | [55.3,62.9] | 14.2 | [11.6,17.3] | 54.9 | [50.6,59.2] |
| **Region of residence** |  | <0.001 |  | <0.001 |  | <0.001 |  | <0.001 |  | 0.103 |
| Kachin | 73.7 | [61.4,83.1] | 37.5 | [26.0,50.7] | 39.3 | [29.4,50.1] | 18.0 | [10.2,29.9] | 62.5 | [52.4,71.6] |
| Kayah | 79.2 | [69.8,86.2] | 21.6 | [13.2,33.3] | 74.1 | [63.1,82.8] | 14.6 | [7.0,27.9] | 55.0 | [43.1,66.4] |
| Kayin | 84.7 | [74.5,91.2] | 17.8 | [9.9,30.0] | 37.8 | [27.6,49.2] | 6.4 | [2.7,14.1] | 50.6 | [40.8,60.3] |
| Chin | 87.8 | [81.2,92.4] | 11.8 | [5.4,23.7] | 55.4 | [46.4,64.1] | 6.4 | [2.3,16.5] | 49.8 | [36.0,63.7] |
| Sagaing | 94.5 | [88.2,97.5] | 9.5 | [5.2,16.8] | 58.6 | [46.0,70.2] | 6.0 | [2.3,14.5] | 57.9 | [46.6,68.4] |
| Taninthayi | 85 | [76.1,91.0] | 19.1 | [12.6,27.9] | 62.6 | [45.8,76.8] | 13.2 | [7.7,21.9] | 57.8 | [44.2,70.3] |
| Bago | 87.3 | [77.5,93.2] | 28.8 | [20.8,38.3] | 60.8 | [50.4,70.3] | 20.3 | [13.3,29.8] | 59.3 | [47.6,70.1] |
| Magway | 95 | [84.3,98.5] | 32.3 | [22.1,44.4] | 78.1 | [65.6,87.0] | 24.2 | [15.4,36.0] | 62.7 | [50.2,73.6] |
| Mandalay | 80.6 | [68.2,89.0] | 46.8 | [36.3,57.6] | 80.6 | [71.8,87.1] | 32.3 | [23.5,42.5] | 67.4 | [55.7,77.3] |
| Mon | 86.4 | [70.3,94.4] | 14.1 | [7.2,25.7] | 60.5 | [43.3,75.4] | 6.3 | [2.6,14.9] | 51.2 | [40.5,61.7] |
| Rakhine | 88.5 | [80.1,93.6] | 17.0 | [10.6,26.1] | 34.5 | [26.6,43.4] | 7.2 | [3.2,15.6] | 54.3 | [43.7,64.4] |
| Yangon | 90.2 | [83.1,94.5] | 14.1 | [7.4,25.0] | 40.5 | [30.2,51.8] | 11.3 | [5.3,22.5] | 67.6 | [55.3,77.8] |
| Shan | 65.7 | [53.9,75.8] | 30.8 | [19.0,45.9] | 63.4 | [53.5,72.3] | 17.1 | [9.8,28.2] | 46.2 | [33.7,59.3] |
| Ayeyarwaddy | 87.5 | [77.5,93.4] | 20.5 | [13.9,29.3] | 51.9 | [41.8,61.8] | 12.9 | [8.2,19.7] | 59.4 | [47.6,70.2] |
| Naypyitaw | 93.9 | [84.3,97.8] | 41.1 | [30.4,52.8] | 73.0 | [57.3,84.5] | 36.6 | [25.5,49.4] | 71.4 | [57.2,82.3] |
| **Family members** |  | <0.001 |  | 0.053 |  | 0.709 |  | 0.024 |  | 0.305 |
| <5 | 90.3 | [86.0,93.4] | 26.5 | [21.6,32.1] | 56.2 | [50.3,62.0] | 19.9 | [15.6,25.0] | 58.0 | [51.8,64.0] |
| 5-6 | 85.6 | [81.2,89.0] | 20.4 | [16.2,25.5] | 59.3 | [54.0,64.4] | 11.4 | [7.9,16.1] | 56.1 | [50.3,61.7] |
| >6 | 77.8 | [72.2,82.5] | 28.5 | [23.1,34.6] | 56.8 | [50.7,62.6] | 17.6 | [13.5,22.6] | 62.0 | [56.3,67.3] |
| **Wealth Index** |  | 0.002 |  | <0.001 |  | 0.407 |  | 0.01 |  | <0.001 |
| Poorest | 88.3 | [82.6,92.2] | 18.5 | [13.8,24.4] | 54.1 | [47.8,60.4] | 10.8 | [7.6,15.2] | 51.3 | [44.3,58.2] |
| Poorer | 87.4 | [81.7,91.5] | 18.3 | [13.5,24.3] | 54.6 | [47.8,61.2] | 13.6 | [9.3,19.3] | 57.9 | [50.8,64.7] |
| Middle | 89.2 | [83.0,93.3] | 22.7 | [17.2,29.4] | 62.4 | [54.5,69.6] | 15.6 | [11.0,21.5] | 51.1 | [43.5,58.6] |
| Richer | 79.3 | [72.7,84.6] | 31.7 | [24.0,40.6] | 59.5 | [51.4,67.1] | 20.8 | [14.8,28.2] | 66.9 | [58.4,74.4] |
| Richest | 75.1 | [66.2,82.2] | 38.7 | [30.8,47.1] | 60.2 | [52.0,67.9] | 22.3 | [16.5,29.3] | 70.1 | [61.7,77.3] |
| **Total** | **84.5** | **[81.7,86.9]** | **24.8** | **[21.7,28.2]** | **57.6** | **[54.3,60.8]** | **15.9** | **[13.5,18.6]** | **58.5** | **[54.8,62.2]** |
